# Draft Genome Sequence of *Pseudoalteromonas* sp. Strain PLSV, an Ulvan-Degrading Bacterium

**DOI:** 10.1128/genomeA.01257-14

**Published:** 2014-12-11

**Authors:** Moran Kopel, William Helbert, Bernard Henrissat, Tirza Doniger, Ehud Banin

**Affiliations:** a The Institute for Nanotechnology and Advanced Materials, Bar-Ilan University, Ramat Gan, Israel; b Mina and Everard Goodman Faculty of Life Sciences, Bar-Ilan University, Ramat Gan, Israel; c Centre de Recherches sur les Macromolécules Végétales, UPR-CNRS 5301, Université Joseph Fourier and Institut de Chimie Moléculaire de Grenoble (ICMG), FR-CNRS 2607, Grenoble, France; d Architecture et Fonction des Macromolécules Biologiques, Centre National de la Recherche Scientiﬁque & Aix-Marseille University, Marseille, France

## Abstract

We present the draft genome sequence of *Pseudoalteromonas* sp. strain PLSV, isolated from the feces of an *Aplysia* sea slug. The addition of the PLSV genome to the existing genomes of three other ulvan-degrading bacterial species will enhance our understanding of ulvan utilization.

## GENOME ANNOUNCEMENT

*Ulva* spp. are green macroalgae used as food or feed (1) and are often involved in the increasing harmful events of eutrophic costal algal blooms known as “green tides” (2). The transformation of this untapped biomass source to compost (3) or the utilization of its rather abundant cell wall polysaccharide, ulvan, is being explored for various biological applications (4). The main building blocks of ulvan are 3-sulfated L-rhamnose, D-glucuronic acid, L-iduronic acid, and D-xylose, mostly found as repeating moieties of ulvanobiouronic acid type A and B disaccharides (5). While the complete pathway for ulvan degradation has yet to be determined, accomplishing this goal might aid in turning these abundant *Ulva* spp. into valuable feedstock. The heterogeneous and complex structure of ulvan requires at least five carbohydrate-cleaving enzymes for its saccharification. Two such enzymes were recently identified and characterized from *Nonlabens ulvanivorans* (6, 7): the ulvan lyase (8), and a member of the glycoside hydrolase family, GH105 (9).

*De novo* sequencing of the ulvan-degrading *Pseudoalteromonas* sp. strain PLSV was performed as described previously (10). The genome was sequenced using the Illumina HiSeq 2000 platform. The ABySS (11) and Velvet (12) *de novo* assemblers were used to assemble the 100 × 2 paired-end library, with an average coverage of approximately 1,933×. A total of 5,256,843 bp, with a G+C content of 44.2%, was assembled into 68 contigs and scaffolds (*N*_50_, 339,956 bp), with a maximum length of 881,179 bp. The RAST server (13) predicted 4,664 coding sequences (3,085 possess annotated functions) belonging to 480 subsystems, including 364 involved in carbohydrate metabolism, 224 in cofactors, vitamins, prosthetic groups, and pigments, 164 in cell wall and capsule, 55 in sulfur metabolism, 162 in stress response, 5 in dormancy and sporulation, 111 in motility and chemotaxis, 34 in iron acquisition and metabolism, 92 in virulence, disease, and defense (out of which 72 code for proteins attributed to resistance to antibiotics and toxic compounds), 162 in membrane transport, 66 in regulation and cell signaling (with 9 attributed to programmed cell death and toxin-antitoxin systems), 6 in regulons, and 8 in phages, prophages, transposable elements, and plasmids. A plasmid partitioning gene, *parA*, was detected, which suggests the occurrence of a plasmid.

This draft genome, along with the additional draft sequences of *N. ulvanivorans* (10) and two *Alteromonas* spp. (14), will allow a better understanding of the utilization and exploitation of the *Ulvales* biomass. A BLAST search of the known ulvan lyase and the GH105 against the draft genome has found no matching sequences. Since the PLSV strain is able to degrade ulvan, it most likely contains genes coding for a distinct set of enzymes that participate in the degradation process. Identifying these enzymes will shed new light on alternative ulvan breakdown pathways.

### Nucleotide sequence accession numbers.

This whole-genome shotgun project has been deposited at DDBJ/EMBL/GenBank under the accession no. JRKG00000000. The version described in this paper is version JRKG01000000.
